# Case report: Discovery of novel *CTNNB1* mutations and comparison of clinical characteristics in two patients with NEDSDV

**DOI:** 10.3389/fgene.2025.1502756

**Published:** 2025-01-28

**Authors:** Eojin Lee, Ja Young Choi, Shin-Seung Yang

**Affiliations:** Department of Rehabilitation Medicine, College of Medicine, Chungnam National University, Daejeon, Republic of Korea

**Keywords:** neurodevelopmental disorder, spasticity, intellectual disability, β-catenin, Wnt signaling pathway, next-generation sequencing

## Abstract

*CTNNB1*, which encodes β-catenin, plays an essential role in the Wnt signaling pathway and regulates cellular homeostasis. Mutations in this gene can lead to neurodevelopmental disorder with spastic diplegia and visual defects (NEDSDV). This study aimed to identify *CTNNB1* mutations in two patients presenting with global developmental delay and compare their distinct phenotypes. Next-generation sequencing (NGS) was performed to detect mutations in *CTNNB1*. Longitudinal clinical observations were conducted to analyze the clinical features of the patients. The first patient was a 7-year-old boy who exhibited symptoms of microcephaly, spasticity, severe amblyopia with retinal detachment, and developmental delay. NGS identified a novel c.1170dupT, p. Ala391CysfsTer4 frameshift variant in *CTNNB1*. The second patient, a 8-year-old girl, had a dysmorphic face, severe global developmental delay, and ataxic gait. NGS revealed a c.1759C > T, p. Arg587Ter nonsense mutation in *CTNNB1*. Both patients shared common NEDSDV features; however, distinct phenotypic variations were observed depending on the type of genomic variant. NGS is crucial for the diagnosis of global developmental delay, particularly when brain magnetic resonance imaging (MRI) results appear normal. The identified novel frameshift variant expands the mutational spectrum of *CTNNB1*.

## 1 Introduction

*CTNNB1* encodes the β-catenin protein, which plays an essential role as part of the cadherin/catenin complex in activating the canonical Wnt signaling pathway and regulating cellular homeostasis (Hamosh et al., 2000). In the central nervous system (CNS), *CTNNB1* mutations can cause defects in synaptic localization and stabilization. Such defects can lead to an imbalance between excitatory and inhibitory signaling, resulting in neurodevelopmental disorders (Bamji et al., 2003; Arredondo et al., 2020). *CTNNB1* is located on chromosome 3p22.1 and is inherited in an autosomal dominant pattern (Ke and Chen, 2020). Neurodevelopmental disorder with spastic diplegia and visual defects (NEDSDV) is a condition associated with the loss of function of *CTNNB1* (OMIM: 116806). Patients with NEDSDV typically present with microcephaly, motor delay, autism spectrum disorder, hypotonia, progressive peripheral spasticity, dysmorphic craniofacial features, and various degrees of visual abnormalities (Kuechler et al., 2015; Kharbanda et al., 2017; Rossetti et al., 2021; Ho et al., 2022) that mimic spastic cerebral palsy. CTNNB1 is also highly expressed in heart, and a recent study has reported patients with varying degrees of cardiac anomalies (Sinibaldi et al., 2023). Although not precisely known, the estimated incidence ranges from 2.6 to 3.2 per 100,000 live births (Ho et al., 1993). As of September 2023, 66 molecular variants of *CTNNB1* associated with NEDSDV have been classified as pathogenic or likely pathogenic according to the ClinVar database. In this study, we identified mutations in *CTNNB1* using next-generation sequencing (NGS) in two patients with developmental delays who presented to the outpatient clinic. One genetic variant has not been previously reported, whereas the other has been reported in Korea (Lee et al., 2022), but exhibits a different phenotype. Through longitudinal clinical observations, we analyzed and compared the distinct clinical features of each patient.

## 2 Case description

**FIGURE 1:**
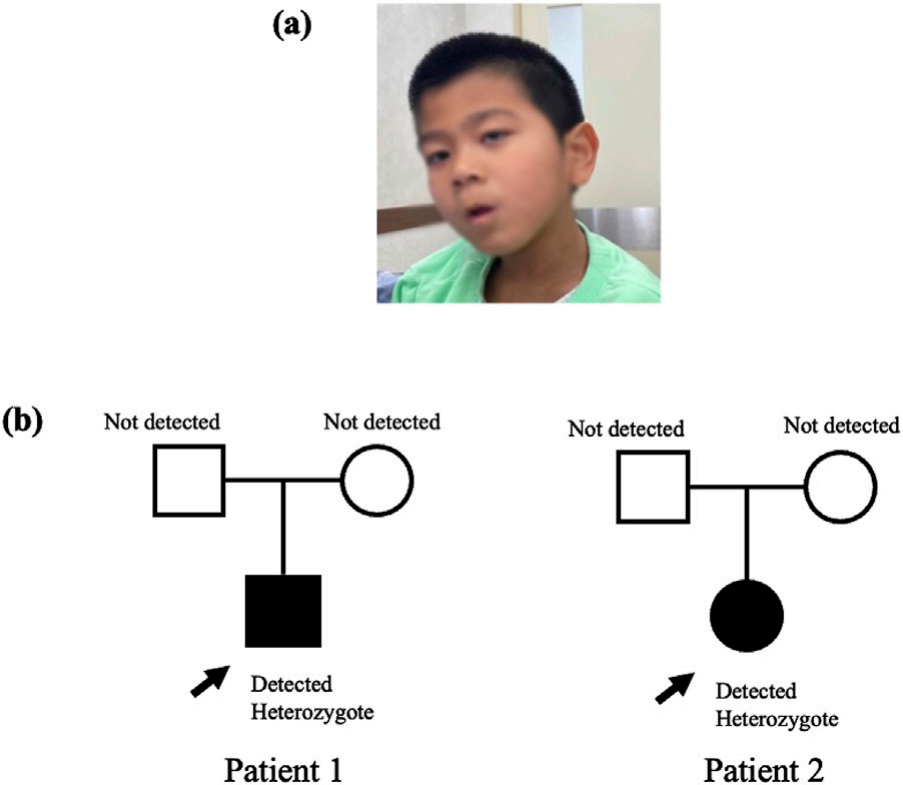
**(A)** Dysmorphic face of Patient 1 **(B)** Pedigree of Patient 1, 2.

**FIGURE 2:**
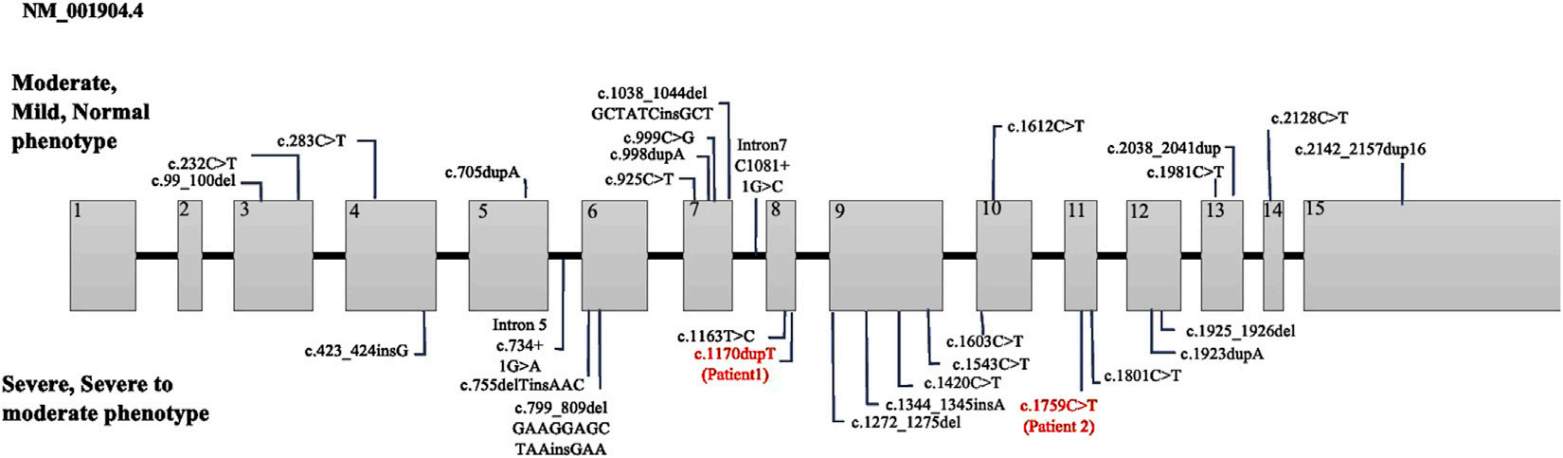
Summary of CTNNB1 variants categorized with phenotype severity. The variants in our study are marked in red.

Patient 1 was a 7-year-old boy born to healthy Korean parents at 40 weeks of gestation with a birth weight of 3.26 kg. There was no family history of genetic disorders, developmental delays, prior admissions to the intensive care unit, or surgical history (Figure 1B). Ultrasonography revealed the presence of microcephaly during pregnancy. At approximately 2 months of age, the symptoms suggested inward deviation of the left eye. Subsequent ophthalmological evaluation at 4 months at our clinic revealed a retinal fold change in the left eye and retinal retraction in the right eye. The patient visited our department for evaluation of motor delay at 9 months of age. The patient achieved the ability to roll over by 5 months and started crawling by 8 months. The patient showed facial dysmorphisms, including microcephaly, arched eyebrows, strabismus, down slanting palpebral fissures, and an elongated facial structure (Figure 1A). In addition, hyperactive deep tendon reflexes were observed in both the knees and ankles. Spasticity in both the upper and lower extremities was graded as Modified Ashworth Scale (MAS) G1+ bilaterally. Ankle clonus was present on both sides. At the age of 3 years, brain magnetic resonance imaging (MRI) and chromosomal microarray analysis were performed to determine the cause of developmental delay and spasticity. However, neither of these studies revealed any abnormal pathological findings. NGS was performed to find the diagnosis, and we identified a heterozygous c.1170dupT variant in *CTNNB1* gene confirmed by Sanger sequencing. The c.1170dupT variant is a null variant (frameshift), fulfilling the guidelines of the American College of Medical Genetics and Genomics (ACMG) and the Association for Molecular Pathology (AMP) criterion PVS1. Parental Sanger sequencing confirmed that this variant occurred as a *de novo* mutation, satisfying the ACMG/AMP criterion PS2. Furthermore, this variant has not been reported in population databases, including gnomAD v2.1, 1000 Genomes, and GINSVPON v1.0, meeting the ACMG/AMP criterion PM2. In conclusion, following the ACMG/AMP guidelines (Richards et al., 2015), this variant was classified as pathogenic. In addition, variants in *CUL7* and *HSPG2* were also identified during the analysis. These variants were found to be inherited from the father and mother, respectively, and were classified as benign variants. To assess the harmfulness of the newly identified CTNNB1 variant, we searched for variants at similar positions in the Global Variome Shared Leiden Open Variation Database and the Decipher database. This allowed us to predict the harmfulness of the new variant located in a region near previously reported pathogenic variant locations within exon 8 (Figure 2). At the age of 5 years, the patient’s weight was 16 kg (below the third percentile) and height was 100 cm (between the third and fifth percentiles). At 6 years of age, the patient demonstrated spastic and ataxic gait patterns requiring an orthosis, such as an ankle-foot orthosis, and walking assistance. His gross motor function measurement (GMFM) total score was 71.99%, and the Berg balance scale (BBS) score was 5/56. At 7 years old, his intelligence quotient (IQ) was below 40, and he had no verbal output although he was able to understand simple commands. Korean-Childhood Autism Rating Scale score was 29 and visual motor integration score was 23.

The patient had several febrile seizures episode exhibiting a generalized tonic-clonic seizure that lasted for 2 min. Subsequent electroencephalography results suggested a mild degree of diffuse encephalopathy. Cardiac function was evaluated through echocardiography and electrocardiography, which revealed no pathological findings.

Patient 2 was a 7-year-old girl who was delivered via cesarean section at 37 weeks with a birth weight of 2.3 kg by health Korean parents. There was no family history of genetic disorders or developmental delays, nor any prior history of surgeries (Figure 1B). The patient visited our clinic at 19 months of age due to concerns about global developmental delay. Simultaneously, the patient developed strabismus and underwent a lateral rectus recession surgery. Physical examination revealed hyperactive deep tendon reflexes in both knees and ankle jerks, and spasticity was observed in both upper and lower extremities. Brain MRI performed showed no abnormal findings. Comprehensive neurometabolic screening, including thyroid function tests, creatine kinase and creatine phosphokinase levels, and rare disease screening yielded normal results. At the age of 4 years, the patient achieved independent walking but exhibited a wide-base ataxic gait and toe clawing. At 6 years of age, the patient was able to speak with three words. At 7 years of age, NGS analysis identified a pathogenic heterozygous c.1759C > T, p. Arg587Ter nonsense variant in *CTNNB1* indicating that the 587th amino acid, arginine was substituted by a termination codon. In addition, missense mutation variants were detected in the DCTN1 and CAPN3 genes. Parental segregation studies revealed that the *CTNNB1* and *CAPN3* variants were confirmed as *de novo* mutations, whereas the *DCTN1* variant was inherited from the father. Based on the guidelines of the ACMG/AMP, the CTNNB1 mutation was classified as a pathogenic variant, and the CAPN3 gene mutation was classified as a variant of uncertain significance (VOUS).

By the age of 9, her receptive language was assessed at the level of 48 months, while her pragmatic language was at the level of 36 months. The BBS was 25/56, and the GMFM score was 68.95%. Additionally, her IQ was measured as 56, and her Social Quotient (SQ) was 53. The Preschool Receptive-Expressive Language Scale indicated that her receptive language was equivalent to a 41-month-old, while her pragmatic language was at the level of a 35-month-old.

## 3 Discussion

*CTNNB1* encodes β-catenin, a protein crucial for brain development and synaptic connections between neurons. In addition, β-catenin regulates gene expression to ensure proper developmental protein production. Mutations in *CTNNB1* disrupt β-catenin production, leading to CTNNB1-syndrome, which manifests as various neurodevelopmental issues owing to impaired signaling pathways. In 2012, initial cases of CTNNB1-syndrome were reported, suggesting the possibility of numerous undiagnosed instances of this condition present in the population. Genetic testing can identify the genetic etiology in approximately 40% of cerebral palsy cases, and recent studies have identified *CTNNB1* as the most prevalent cause of misdiagnosis of cerebral palsy (Jin et al., 2020; Moreno-De-Luca et al., 2021).

This study describes two patients diagnosed with NEDSDV. Both patients presented with common clinical features of NEDSDV, including typical dysmorphic features, neurodevelopmental delays, and vision problem. Furthermore, they displayed hyperactive deep tendon reflexes in both the knee jerk and ankle jerk tests and spasticity was observed in the upper and lower extremities. Brain MRI did not reveal any significant abnormalities in either patient.

Both patients have participated in a multidisciplinary rehabilitation program, which includes physical therapy, occupational therapy, and speech therapy as part of an outpatient program, along with special education. Despite these interventions, Patient 1 could not walk independently due to severe spasticity in both lower limbs and requires the use of ankle-foot orthoses (AFO) yet. In contrast, Patient 2 experienced less severe spasticity than Patient 1 and could walk independently. However, frequent falls were noted due to balance difficulties.

In terms of cognitive and language development, Patient 2 has made progress and was able to construct sentences consisting of three words. This patient had an IQ of 56 indicating mild intellectual disability. Meanwhile, Patient 1 exhibited profound intellectual disability with an IQ below 40, which reflected severe cognitive and developmental delays compared to patient 2.

Miroševič et al. (2022) investigated the genotype-phenotype correlation in CTNNB1 syndrome, highlighting a certain relationship between phenotype severity and both the mutation location and type. The critical interaction surface of β-catenin was extensive, spanning armadillo repeats 3 to 9, which are encoded by exons 5 to 10. Mutations within this region were particularly detrimental as they disrupt β-catenin’s structural integrity and its ability to mediate essential cellular functions, including transcriptional regulation and cellular adhesion. In our study, Patient 1 exhibits a frameshift mutation in exon 8, which aligns with the severe phenotype described by Miroševič et al. (Table 1). The disrupted armadillo repeat region likely results in significant functional loss, explaining the patient’s profound motor and cognitive impairments. On the other hand, Patient 2 has a nonsense mutation in exon 11 within the armadillo repeat region, but at a position where the structural and functional impacts may be less critical. This corresponds to the moderate phenotype observed in the patient 2, with preserved motor and cognitive functions to a greater extent. These observations support the critical role of mutation location and type in determining clinical severity in CTNNB1-related disorders.

**TABLE 1:** CTNNB1 mutations and clinical characteristics in patients with NEVSDV categorized by phenotype severity.

Clinical characteristics	Gender/Age (y)	HGVSc/HGVSp	Variant type, exon	Inheritance	Last motor status	Last language status	Ophthalmologic features	Behavior and IQ	Brain MRI/Seizure
Severe phenotype									
Kuechler et al. (2015): Patient 4	F/15	c.755delTinsAAC/p.Leu252*	Nonsense, 6/15	*De novo*	Walks independently at 10 years	2 words	Strabismus, hyperopia	Auto-aggressive behavior, stereotypic movements, short eye contact; severe IQ	WNL/NO
Kharbanda et al. (2017): Patient 8	F/7	c.799_809del GAAGGAGC TAAinsGAA/p.Gly268TrpfsTer5	Frameshift, 6/15	*De novo*	Walks independently at 3 years, broad based gait	No words	NA	Autism, ID	WNL/NO
Patient 1 of this study	M/6	c.1170dupT/p.Ala391CysfsTer4	Frameshift, 8/15	*De novo*	Walks with support, spastic and ataxic gait	No words, understand simple command	Strabismus, both retinopathy retinal retraction, avascular retina (Rt), blindness (Lt)	Autism, IQ < 40	WNL/YES
Severe to moderate phenotype									
Kuechler et al. (2015): Patient 5	F/5	c.423_424insG/p.Tyr142Valfs*4	Frameshift, 4/15	*De novo*	Walks with support, Hypotonic, ataxic gait	30 words, sign language	Strabismus	Repetitive movements, ID	WNL/NO
Kuechler et al. (2015): Patient 11	F/6	c.1163T > C/p.Leu388Pro	Missense, 8/15	*De novo*	Walks independently at 30 months old	20 words at 4 years old	NA	ID	WNL/NO
Kuechler et al. (2015): Patient 15	F/3	c.1272_1275del/p.Ser425Thrfs*11	Frameshift, 9/15	*De novo*	Unable to walk	Babbles now, some words are understand- able	Strabismus	Very happy and friendly, low frustration tolerance	WNL/NO
Kharbanda et al. (2017): Patient 1	M/6	c.1801C > T/p.Gln601Ter	Nonsense, 11/15	*De novo*	Unable to walk	Says Mom and dad with under- standing, uses Makaton, points to body parts	FEVR	Occasional temper; can bite others and self; repetitive movements	WNL/NO
Lee et al. (2022): patient 6	F/10	c.1759C > T/p.Arg587Ter	Nonsense, 11/15	*De novo*	Walks alone, tiptoe and ataxic gait	No words	Rt ptosis, strabismus	aggressive behavior, impulsivity, head stereotypy, bruxism, and breathing irregularities	WNL/NO
Moderate phenotype									
Kharbanda et al. (2017): patient 5	F/11	c.1038_1044del GCTATCTinsGCT/p.Val349AlafsTer9	Frameshift, 7/15	*De novo*	Walks independently at 42 months, ataxic gait	Single words at age 5 years, talks in sentences at age 11 years	Strabismus, hypermetropia	Stereotypies (clapping repeatedly, temper tantrums, aggressive to family)	WNL/NO
Kharbanda et al. (2017): patient 9	F/4	c.1612C > T/p.Gln538Ter	Nonsense, 10/15	*De novo*	Walks independently at 3 years	First words at 3.4 years	Strabismus	Autism	WNL/NO
Patient 2 of this study	F/7	c.1759C > T/p.Arg587Ter	Nonsense, 11/15	*De novo*	Walk alone at 4 years old, wide-base ataxic gait	3 words sentence at 6 years old	Strabismus	IQ 56	WNL/NO
Mild phenotype									
Kuechler et al. (2015): patient 6	F/13	c.2038_2041dup / p.Ser681*	Nonsense, 13/15	*De novo*	Walk alone at 1.5 years	Mild, full sentences, but delayed	Strabismus, myopia	Social, autism, aggressive behavior, ADHD	WNL/NO
Kharbanda et al. (2017): patient 3	F/9	c.1981C > T/p.Arg661Ter	Nonsense, 13/15	*De novo*	Walk alone at 2.5 years	First words at 3.4 years	NA	Obsessional behavior; dyspraxia	WNL/NO
Normal phenotype									
Panagiotou et al. (2017)	M	c.2128C > T/p.Arg710Cys	Missense	Autosomal recessive	Normal	Normal	FEVR	Normal	WNL/NO

Interestingly, significant phenotypic differences were observed when comparing our Patient 2 with the case reported by Lee et al., who carried the same c.1759C > T, p. Arg587Ter mutation. In terms of language development, the patient described by Lee et al. did not produce any words whereas our patient 2 was able to construct three-word sentences by the age of 6. In addition, the patient reported by Lee et al. exhibited aggressive behavior, impulsivity, head stereotypy, bruxism, and breathing irregularities, which are autistic features unlike our patient 2 who did not show such behavior symptoms.

These findings highlight the phenotypic variability associated with the same CTNNB1 mutation. Such differences may be attributable to additional factors beyond the primary genetic mutation, including genetic modifiers, epigenetic influences and environmental conditions that may affect the Wnt/β-catenin signaling pathway. Further research is necessary to investigate these factors and their potential interactions with CTNNB1 mutations in shaping phenotypic outcomes.

Treatment of CTNNB1-syndrome focuses on managing specific clinical manifestations, as no standardized protocols exist. A multidisciplinary approach involving a team of specialists is essential for individualized care. Key approaches include regular assessments and treatment adjustments to meet the changing needs of patients. This may involve various specialists, such as neurologists, ophthalmologists, and rehabilitation therapists. Interventions often include physical, occupational, and speech therapies, the use of assistive devices, and potentially surgical options for severe cases. Continuous monitoring and adaptive strategies are essential for effectively managing the diverse clinical manifestations of CTNNB1 Syndrome (Ho et al., 1993). Recent clinical and preclinical studies have demonstrated that modulation of the Wnt/β-catenin signaling pathway using pharmacological agents such as lithium, SB216763, and sulindac, or by targeting the transcriptional factor with a PPARγ agonist, may offer therapeutic benefits for neurodevelopmental delays (Zhuang et al., 2023).

The limitations of this study include insufficient follow-up duration and lack of longitudinal assessments of visual field problem.

## 4 Conclusion

This study highlighted the significant phenotypic variability associated with CTNNB1 mutations, as demonstrated by two patients with NEDSDV. Despite sharing common clinical features such as dysmorphic traits, neurodevelopmental delays, and spasticity, the differences in cognitive and motor outcomes emphasize the influence of mutation location and type on phenotype severity. The phenotypic differences at the same mutation location highlight the complexity of CTNNB1 syndrome and suggest the involvement of additional factors, such as genetic modifiers and epigenetic mechanisms. This study emphasizes the need for further research to investigate the mechanisms underlying this variability, particularly the modulation of the Wnt/β-catenin signaling pathway.

## Data Availability

The original contributions presented in the study are included in the article/supplementary material, further inquiries can be directed to the corresponding author.
